# The glucagon-like peptide-1 (GLP-1) analogue semaglutide reduces alcohol drinking and modulates central GABA neurotransmission

**DOI:** 10.1172/jci.insight.170671

**Published:** 2023-06-22

**Authors:** Vicky Chuong, Mehdi Farokhnia, Sophia Khom, Claire L. Pince, Sophie K. Elvig, Roman Vlkolinsky, Renata C.N. Marchette, George F. Koob, Marisa Roberto, Leandro F. Vendruscolo, Lorenzo Leggio

**Affiliations:** 1 Clinical Psychoneuroendocrinology and Neuropsychopharmacology Section, Translational Addiction Medicine Branch, National Institute on Drug Abuse Intramural Research Program (NIDA IRP) and National Institute on Alcohol Abuse and Alcoholism Division of Intramural Clinical and Biological Research (NIAAA DICBR), NIH, Baltimore and Bethesda, Maryland, USA.; 2 Neurobiology of Addiction Section, NIDA IRP, NIH, Baltimore, Maryland, USA.; 3 Department of Molecular Medicine, The Scripps Research Institute, La Jolla, California, USA.; 4 Department of Pharmaceutical Sciences, University of Vienna, Vienna, Austria.; 5 Stress and Addiction Neuroscience Unit, NIDA IRP and National Institute on Alcohol Abuse and Alcoholism Division of Intramural Clinical and Biological Research, National Institutes of Health, Baltimore, Maryland, USA.

**Keywords:** Endocrinology, Neuroscience, Addiction

## Abstract

Growing evidence indicates that the glucagon-like peptide-1 (GLP-1) system is involved in the neurobiology of addictive behaviors, and GLP-1 analogues may be used for the treatment of alcohol use disorder (AUD). Here, we examined the effects of semaglutide, a long-acting GLP-1 analogue, on biobehavioral correlates of alcohol use in rodents. A drinking-in-the-dark procedure was used to test the effects of semaglutide on binge-like drinking in male and female mice. We also tested the effects of semaglutide on binge-like and dependence-induced alcohol drinking in male and female rats, as well as acute effects of semaglutide on spontaneous inhibitory postsynaptic currents (sIPSCs) from central amygdala (CeA) and infralimbic cortex (ILC) neurons. Semaglutide dose-dependently reduced binge-like alcohol drinking in mice; a similar effect was observed on the intake of other caloric/noncaloric solutions. Semaglutide also reduced binge-like and dependence-induced alcohol drinking in rats. Semaglutide increased sIPSC frequency in CeA and ILC neurons from alcohol-naive rats, suggesting enhanced GABA release, but had no overall effect on GABA transmission in alcohol-dependent rats. In conclusion, the GLP-1 analogue semaglutide decreased alcohol intake across different drinking models and species and modulated central GABA neurotransmission, providing support for clinical testing of semaglutide as a potentially novel pharmacotherapy for AUD.

## Introduction

Alcohol use disorder (AUD) is a chronic, relapsing disorder and one of the leading causes of preventable death worldwide. Despite the high morbidity and mortality associated with AUD, there are only a few approved effective pharmacotherapies, and they are underutilized. Therefore, there is a critical need to identify and develop additional medications for AUD (1). Growing evidence indicates overlapping neurobiological mechanisms that underlie pathological overeating and addictive behaviors (2, 3). Accordingly, systems that control appetite and feeding are under investigation as potential pharmacotherapeutic targets for AUD (4, 5). One such target is the glucagon-like peptide-1 (GLP-1), an incretin hormone and neuropeptide involved in regulating appetite, food intake, and metabolism (6).

GLP-1 is a 30 aa peptide produced by cleavage of preproglucagon in intestinal endocrine L cells and in the nucleus tractus solitarius (NTS) neurons (7–9). GLP-1 exerts insulinotropic effects in hyperglycemic states and decreases food intake through both central and peripheral mechanisms (10, 11). Growing evidence also suggests that GLP-1 modulates stress, mood, cognition, and reward processing (12–16). Administration of GLP-1 itself or GLP-1 analogues in rodents has been shown to reduce the rewarding effects of addictive drugs, including stimulants, opioids, nicotine, and alcohol (6, 17). The G protein–coupled GLP-1 receptors (GLP-1Rs) are widely expressed in peripheral organs such as the pancreas, liver, and gastrointestinal tract as well as brain regions involved in appetitive behaviors and reward such as hypothalamus, nucleus accumbens, and ventral tegmental area (18–21). GLP-1Rs are also highly expressed in the central nucleus of the amygdala (CeA) and the infralimbic cortex (ILC) (22, 23). GLP-1R expression in these key reward- and stress-related brain regions may contribute to food (24-27) and alcohol (28–30) seeking and consumption. Of note, GABAergic transmission is elevated in the CeA following both acute and chronic alcohol exposure, representing critical neuroadaptations in the transition to dependence (28, 31–33). Additionally, glutamatergic and GABAergic signaling in the ILC contributes to inhibitory control over alcohol seeking and relapse (29, 34–37). Although GLP-1R stimulation has been shown to modulate GABAergic signaling in the hippocampus and NTS (38–40), the effects of GLP-1R agonism on GABAergic synapses in the CeA and ILC, especially in the context of alcohol drinking, are unknown.

Because GLP-1 has a short half-life of approximately 2 minutes, GLP-1 analogues with longer half-lives have been developed and are now widely used for the treatment of type 2 diabetes mellitus and obesity (41–43). Previous studies show that administration of GLP-1 analogues, including exenatide (exendin-4), dulaglutide, and liraglutide in mice, rats, and nonhuman primates, suppressed outcomes related to alcohol reward, including alcohol-induced dopamine release in the nucleus accumbens, conditioned place preference for alcohol, and alcohol self-administration (6, 17). We recently tested the effects of 2 long-acting GLP-1 analogues, liraglutide and semaglutide in male Wistar rats and found that both drugs reduced voluntary alcohol intake in an intermittent-access, 2-bottle choice test. Unlike liraglutide, semaglutide also reduced alcohol preference without reducing water intake (44). Compared with other selective GLP-1 analogues, semaglutide is more potent and has higher affinity for GLP-1R, resulting in greater weight loss and glucose-lowering properties (45–47). The long half-life of semaglutide (approximately 7.5 hours in mice, 12 hours in rats, and 183 hours in humans) makes it suitable for once-weekly administration in humans (43, 48–50). In addition to the s.c. formulation, semaglutide is currently the only selective GLP-1 analogue with an FDA-approved oral formulation (51). These factors make semaglutide an ideal GLP-1 analogue for clinical translation in individuals with AUD. However, additional information is needed on whether and how semaglutide may influence biobehavioral correlates of alcohol drinking and dependence.

In the present study, we examined different doses of semaglutide in a binge-like drinking procedure in mice, a binge-like drinking procedure in rats, and a dependence model in rats. To investigate the specificity (or lack) of semaglutide’s effect in reducing alcohol intake, we also tested the effects of semaglutide on locomotion, motor coordination, blood alcohol levels (BALs), and the consumption of other solutions not containing alcohol. Finally, electrophysiological recordings were performed in the CeA and ILC of alcohol-naive and alcohol-dependent rats to assess the effects of an acute application of semaglutide on GABA_A_ receptor–mediated synaptic transmission. We hypothesized that semaglutide would decrease the consumption of alcohol and caloric/palatable solutions, without changing the consumption of noncaloric solutions, spontaneous locomotion, motor coordination, and BALs. We also hypothesized that semaglutide would normalize alcohol-induced dysregulation in central GABA neurotransmission.

## Results

### Effects of semaglutide on the consumption of sweet and unsweet alcohol solutions and a sweet solution not containing alcohol.

**Figure 1:**
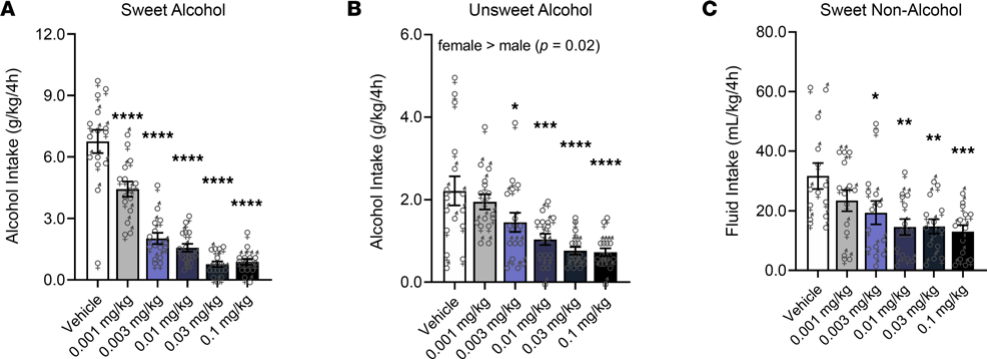
Semaglutide reduces binge-like alcohol drinking in mice. (**A**) Semaglutide reduced alcohol intake (g/kg of body weight) in mice drinking sweet alcohol. Males (*n* = 8); females (*n* = 7). (**B**) Semaglutide reduced alcohol intake (g/kg of body weight) in mice drinking unsweet alcohol; female mice drank significantly more alcohol than males. Males (*n* = 8); females (*n* = 8). (**C**) Semaglutide reduced fluid intake (mL/kg of body weight) in mice drinking a sweet solution not containing alcohol. Males (*n* = 8); females (*n* = 6). Separate cohorts of mice were used to test the effects of semaglutide on the consumption of each drinking solution. Data are expressed as mean ± SEM and were analyzed using 2-way repeated-measures ANOVA. *P < 0.05, **P < 0.01, ***P < 0.001, ****P < 0.0001 versus vehicle. Individual values are presented for males (♂) and females (♀).

For mice drinking sweetened (sweet) alcohol, a main effect of Dose (*F*_5,65_ = 51.81, *P* < 0.0001) was found; semaglutide at all doses (*P* < 0.0001), compared with vehicle, reduced intake. There was no main effect of Sex or Dose × Sex interaction. Male and female data were combined for visualization, but the individual data points are depicted by sex-specific symbols (Figure 1A).

For mice drinking unsweetened (unsweet) alcohol, a main effect of Dose (*F*_5,70_ = 9.12, *P* < 0.0001) was found; semaglutide at 0.003 mg/kg (*P* = 0.05), 0.01 mg/kg (*P* = 0.0007), 0.03 mg/kg (*P* < 0.0001), and 0.1 mg/kg (*P* < 0.0001), compared with vehicle, reduced intake. A main effect of Sex (*F*_1,14_ = 7.66, *P* = 0.02; female > male), but no Dose × Sex interaction, was also observed (Figure 1B).

For mice drinking a sweet caloric solution not containing alcohol (glucose + saccharin), a main effect of Dose (*F*_5,65_ = 5.53, *P* = 0.0003) was found; semaglutide at 0.003 mg/kg (*P* = 0.021), 0.01 mg/kg (*P* = 0.001), 0.03 mg/kg (*P* = 0.002), and 0.1 mg/kg (*P* = 0.0007), compared with vehicle, reduced intake. There was no main effect of Sex or Dose × Sex interaction (Figure 1C).

### Effects of semaglutide on the consumption of other drinking solutions and chow/water intake in mice.

**Figure 2:**
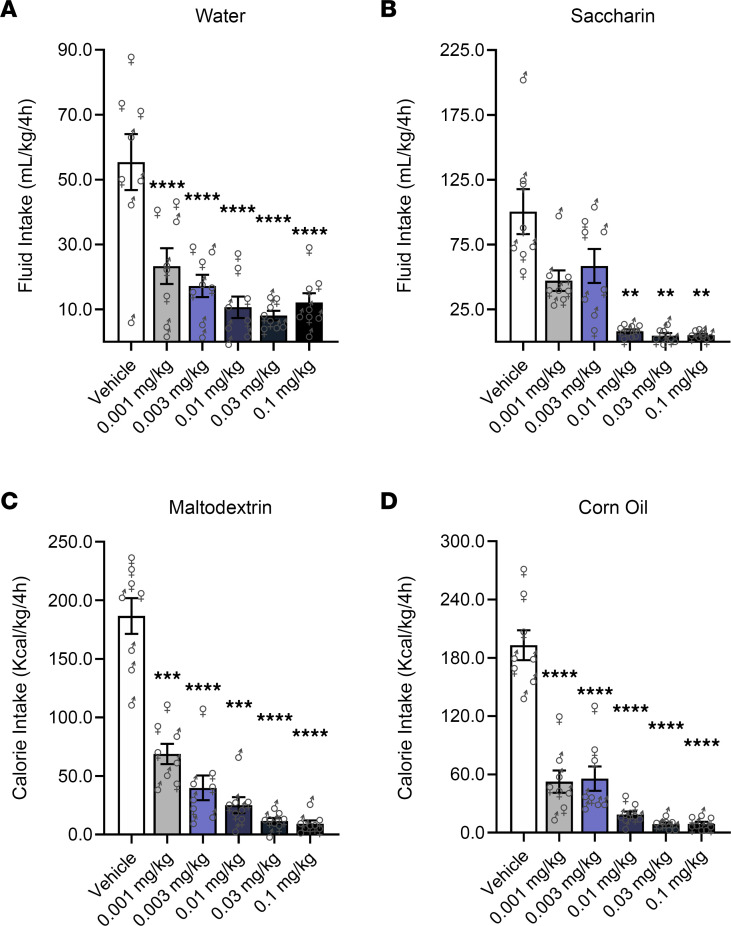
Semaglutide reduces drinking of noncaloric and caloric solutions not containing alcohol in mice. (**A** and **B**) Semaglutide reduced fluid intake (mL/kg of body weight) in mice drinking water or a saccharin-sweetened noncaloric solution. (**C** and **D**) Semaglutide reduced calorie intake (Kcal/kg of body weight) in mice drinking an unsweet carbohydrate (maltodextrin) solution or an unsweet fat (corn oil) emulsion. Separate cohorts of mice were used to test the effects of semaglutide on the consumption of each drinking solution (*n* = 8, 4 per sex, per condition). Data are expressed as mean ± SEM and were analyzed using 1-way repeated-measures ANOVA. **P < 0.01, ***P < 0.001, ****P < 0.0001 versus vehicle. Individual values are presented for males (♂) and females (♀).

For mice drinking water, a main effect of Dose (*F*_5,35_ = 18.64, *P* < 0.0001) was found; semaglutide at all doses (*P* < 0.0001), compared with vehicle, reduced intake (Figure 2A). For mice drinking a sweet noncaloric solution (saccharin), a main effect of Dose (*F*_5,35_ = 18.02, *P* < 0.0001) was found; semaglutide at 0.01 mg/kg (*P* = 0.005), 0.03 mg/kg (*P* = 0.002), and 0.1 mg/kg (*P* = 0.003), compared with vehicle, reduced intake (Figure 2B).

For mice drinking caloric solutions, either an unsweet carbohydrate (maltodextrin) solution or an unsweet fat (corn oil) emulsion, a main effect of Dose (maltodextrin: *F*_5,35_ = 57.14, *P* < 0.0001; corn oil: *F*_5,35_ = 78.43, *P* < 0.0001) was found; semaglutide at all doses (*P* < 0.001), compared with vehicle, reduced intake (Figure 2, C and D).

Chow and water intake were examined in mice that were previously drinking unsweet alcohol. For chow intake, a main effect of Dose (*F*_5,70_ = 36.7, *P* < 0.0001) was found; semaglutide at all doses (*P* < 0.0001) except 0.001 mg/kg, compared with vehicle, reduced chow intake. For water intake, a main effect of Dose (*F*_5,70_ = 23.91, *P* < 0.0001) was found; semaglutide at all doses (*P* < 0.001), compared with vehicle, reduced water intake (Supplemental Table 1; supplemental material available online with this article; https://doi.org/10.1172/jci.insight.170671DS1).

### Effects of semaglutide on motor coordination and BALs in mice.

Saline-treated mice were tested on the rotarod to determine whether semaglutide per se affects motor coordination (saline condition; Supplemental Figure 1A). Although a significant Dose effect (*F_2,84_* = 10.96, *P* < 0.0001; 0.01 mg/kg < 0 and 0.1 mg/kg) was found, semaglutide did not change motor coordination, compared with baseline (i.e., no Dose × Time interaction). The main effect of Time was not significant.

We also evaluated the effects of semaglutide on alcohol-induced ataxia (Supplemental Figure 1B). A main effect of Time (*F_5,195_* = 187.0, *P* < 0.0001) was found, indicating that alcohol induced motor incoordination, and this effect ameliorated over time. However, no Dose or Dose × Time interaction was shown, indicating that semaglutide did not influence alcohol-induced ataxia.

Blood was collected 30 minutes and 90 minutes after alcohol injection, immediately following the rotarod testing, to measure BALs (Supplemental Figure 1C). A main effect of Time (*F_1,39_* = 231.9, *P* < 0.0001) was found, indicating that BALs were lower at 90 than 30 minutes. Although the Dose × Time interaction was significant (*F_2,39_* = 94.5, *P* = 0.02), post hoc comparison did not show any differences. The main effect of Dose was not significant.

Effects of semaglutide on spontaneous locomotion in mice were evaluated by measuring the distance traveled in the circular corridor test (Supplemental Figure 1D). A main effect of Dose (*F*_2,14_ = 37.37, *P* < 0.0001) was found; semaglutide at 0.1 mg/kg (*P* < 0.0001), compared with vehicle, decreased locomotion.

### Effects of semaglutide on alcohol and water self-administration in rats.

**Figure 3:**
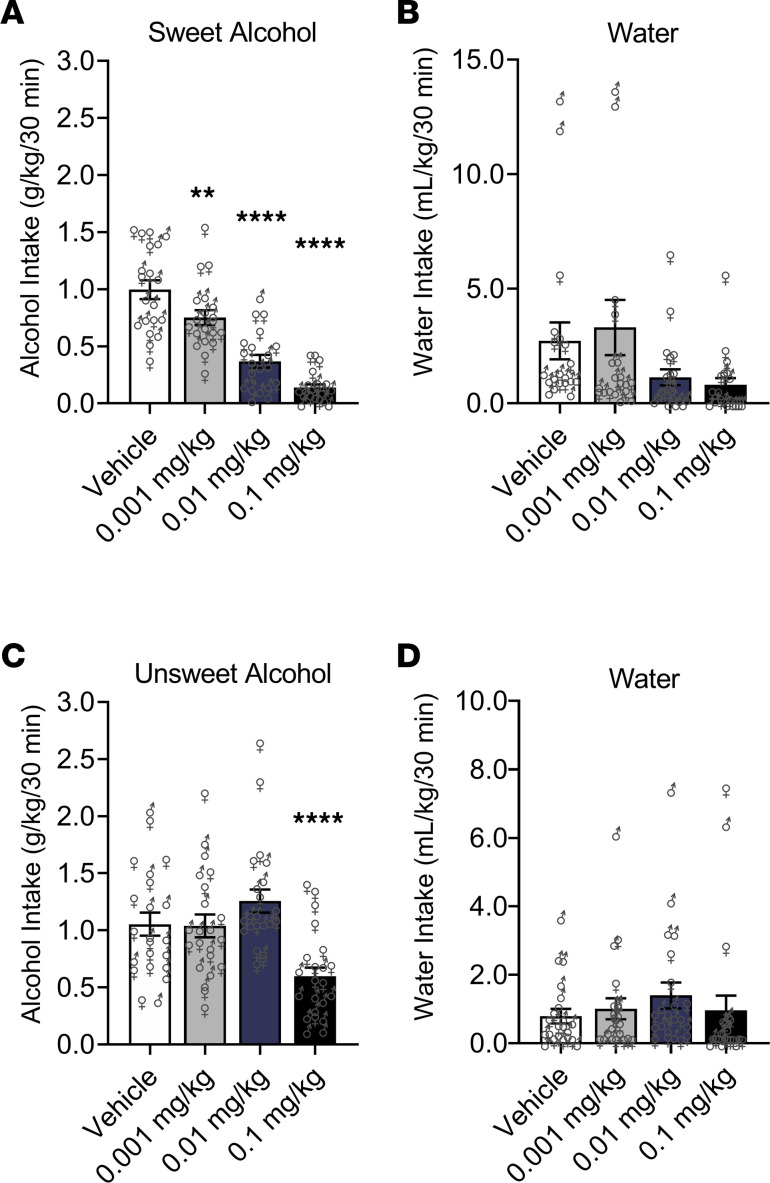
Semaglutide reduces operant alcohol self-administration in rats. (**A**) Semaglutide dose-dependently reduced sweet alcohol self-administration (binge-like drinking) in rats. (**B**) Semaglutide did not reduce water self-administration in nondependent rats (significant Dose effect, but no significant post hoc differences); female nondependent rats self-administered significantly more water than males. Nondependent males (*n* = 10); nondependent females (*n* = 10). (**C**) Semaglutide only at the highest dose (0.1 mg/kg) reduced unsweet alcohol self-administration (dependence-induced drinking) in rats. (**D**) Semaglutide had no effect on water self-administration in alcohol-dependent rats; male dependent rats self-administered significantly more water than females. Dependent males (*n* = 11); dependent females (*n* = 11). Data are expressed as mean ± SEM and were analyzed using 2-way repeated-measures ANOVA. **P < 0.01, ****P < 0.0001 versus vehicle. Individual values are presented for males (♂) and females (♀).

In nondependent rats, a main effect of Dose (*F*_3,54_ = 57.11, *P* < 0.0001), but no effect of Sex or Dose × Sex interaction, was found for alcohol binge-like drinking. Compared with vehicle, semaglutide at all doses (0.001 mg/kg, *P* < 0.01; 0.01 mg/kg, *P* < 0.0001; 0.1 mg/kg, *P* < 0.0001) reduced self-administration of the sweet alcohol solution (Figure 3A). For water self-administration, a main effect of Dose (*F*_3,54_ = 3.95, *P* = 0.01; post hoc comparisons did not indicate significant differences) and Sex (*F*_1,18_ = 9.33, *P* = 0.007; female > male), but no Dose × Sex interaction, was found (Figure 3B).

In alcohol-dependent rats, a main effect of Dose (*F*_3,60_ = 11.24, *P* < 0.0001), but no effect of Sex or Dose × Sex interaction, was found for dependence-induced drinking. Compared with vehicle, semaglutide at 0.1 mg/kg (*P* = 0.0007) reduced self-administration of the unsweet alcohol solution (Figure 3C). For water self-administration, a main effect of Sex (*F*_1,20_ = 6.91, *P* = 0.01; male > female), but no effect of Dose or Dose × Sex interaction, was found (Figure 3D).

### Effects of semaglutide on spontaneous locomotion in dependent rats.

The distance traveled in the open field was not significantly different under semaglutide (14.26 ± 7.9 m) and vehicle (15.26 ± 2.0 m).

### Effects of alcohol vapor exposure on inhibitory neurotransmission in the CeA and ILC.

As shown in Supplemental Figure 2, A–C, and Supplemental Table 2A, and in line with our previous work (31–33), alcohol vapor exposure significantly elevated GABA_A_ receptor–mediated neurotransmission in the medial subdivision of the CeA, as indicated by significantly increased spontaneous inhibitory postsynaptic current (sIPSC) frequencies (*t* = 2.94, *df* = 31, *P* = 0.006) in alcohol-dependent, compared with alcohol-naive, rats. Other sIPSC characteristics such as amplitudes, rise time, and decay time did not differ between the 2 groups.

As shown in Supplemental Figure 2, D–F, and Supplemental Table 2B, alcohol vapor exposure also significantly elevated GABA_A_ receptor–mediated neurotransmission in pyramidal neurons located in layer 5 of the ILC. Specifically, increased frequencies (*t* = 2.08, *df* = 24, *P* = 0.04) and amplitudes (*t* = 3.19, *df* = 24, *P* = 0.003) of sIPSCs onto ILC neurons were found in alcohol-dependent, compared with alcohol-naive, rats. The sIPSC kinetics (i.e., current rise and decay times) did not differ between the 2 groups. These data indicate that alcohol vapor exposure induces ILC neuroadaptations at both pre- and postsynaptic sites.

### Effects of semaglutide on inhibitory neurotransmission in the CeA and ILC.

**Figure 4:**
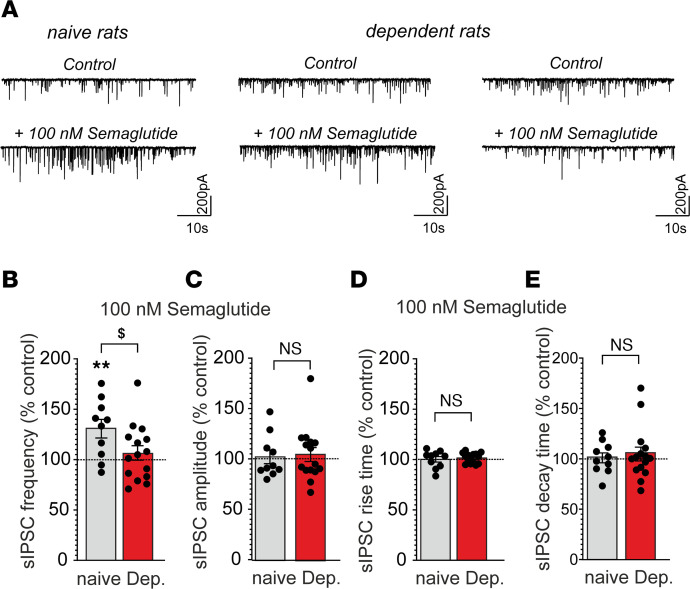
Semaglutide increased GABA transmission in central nucleus of the amygdala (CeA) neurons from alcohol-naive rats but had mixed effects in alcohol-dependent rats. (**A**) Representative spontaneous inhibitory postsynaptic current (sIPSC) traces during baseline control (upper panel) conditions and during superfusion of 100 nM semaglutide (lower panel). (**B**–**E**) Bar charts summarize the effects of semaglutide (100nM) on sIPSC frequencies (**B**), amplitudes (**C**), rise times (**D**), and decay times (**E**) from 10 to 15 neurons from alcohol-naive (gray bars) and alcohol-dependent rats (red bars). Data are expressed as mean ± SEM. Differences between semaglutide and baseline control conditions (dashed lines) were analyzed using 1-sample Student’s *t* tests (**P < 0.01). Differences of semaglutide effects on selected parameters between alcohol-naive and alcohol-dependent rats were analyzed using unpaired Student’s *t* tests (^$^*P* < 0.05). Data were generated from 6 alcohol-naive and 8 alcohol-dependent rats, from 2 separate chronic, intermittent, alcohol vapor exposure cohorts.

In alcohol-naive rats, acute application of semaglutide significantly increased sIPSC frequency in CeA neurons (130.7% ± 9.2%; *t* = 3.33, *df* = 9, *P* = 0.008), without affecting postsynaptic measures (amplitudes, rise time, or decay time), suggesting enhanced GABA release. In contrast, in alcohol-dependent rats, semaglutide overall did not alter any sIPSC parameter. Of note, semaglutide increased GABA release in a subset of CeA neurons and decreased it in another subset (Figure 4).

**Figure 5:**
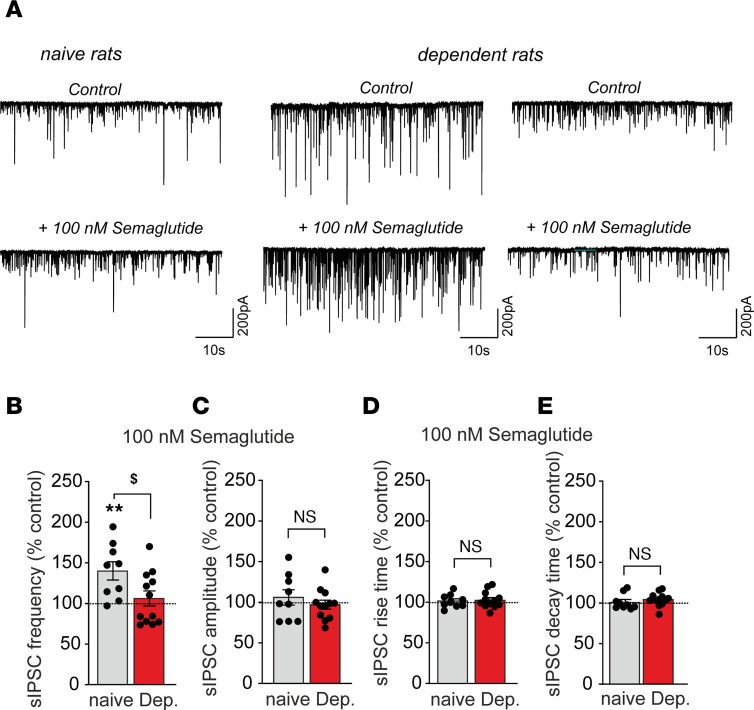
Semaglutide increased GABA transmission in pyramidal neurons in layer 5 of the infralimbic cortex (ILC) from alcohol-naive rats but had mixed effects in alcohol-dependent rats. (**A**) Representative spontaneous inhibitory postsynaptic currents (sIPSC) traces during baseline control (upper panel) conditions and during superfusion of 100 nM semaglutide (lower panel). (**B**–**E**) Bar charts summarize the effects of semaglutide (100nM) on sIPSC frequencies (**B**), amplitudes (**C**), rise times (**D**), and decay times (**E**) from 9 to 12 neurons from alcohol-naive (gray bars) and alcohol-dependent rats (red bars). Data are expressed as mean ± SEM. Differences between semaglutide and baseline control conditions (dashed lines) were analyzed using 1-sample Student’s *t* tests (**P < 0.01). Differences of semaglutide effects on selected parameters between alcohol-naive and alcohol-dependent rats were calculated using unpaired Student’s *t* tests (^$^*P* < 0.05). Data were generated from 5 alcohol-naive and 7 alcohol-dependent rats, from 2 separate chronic, intermittent, alcohol vapor exposure cohorts.

In alcohol-naive rats, acute application of semaglutide significantly increased sIPSC frequency in ILC neurons (140.1% ± 11.2%, *t* = 3.56, *df* = 8, *P* = 0.007), without affecting postsynaptic measures (amplitudes, rise time, or decay time), suggesting enhanced GABA release. In contrast, in alcohol-dependent rats, semaglutide overall did not alter any sIPSC parameter. Similar to the CeA, semaglutide increased GABA release in a subset of ILC neurons and decreased it in another subset (Figure 5).

## Discussion

Growing literature suggests an important role of the GLP-1 system in AUD and the potential for this pharmacological target to be translated to humans, given the increasing use of GLP-1 analogues to treat type 2 diabetes mellitus and/or obesity. Most of the work on GLP-1 in the alcohol field has been done with the prototype drug exenatide and, more recently, with liraglutide and dulaglutide, but literature is scarce on the potential impact of semaglutide, the newest FDA-approved GLP-1 analogue with high translational advantages, on alcohol-related outcomes (17). In a preliminary set of experiments, we previously showed that both liraglutide and semaglutide reduced alcohol intake in Wistar rats tested on a 2-bottle, free-choice procedure, but only semaglutide reduced alcohol preference; however, this work was limited to nondependent male rats (44). Considering these previous findings, combined with growing literature suggesting that semaglutide has higher GLP-1R binding and greater clinical efficacy than other selective GLP-1 analogues on glucose control and weight loss (43, 45–50), the present work aimed to provide detailed information on the biobehavioral effects of semaglutide in relation to alcohol use in mice and rats of box sexes.

Our findings here demonstrate that semaglutide reduced binge-like alcohol drinking in both mice and rats. This effect was observed in males and females, and no sex differences were detected. Of note, the ability of semaglutide to reduce binge-like alcohol drinking was dose dependent, further supporting a causal role of semaglutide. Binge drinking is a critically concerning pattern in individuals with unhealthy alcohol use and is responsible for significant mortality and morbidity. Binge drinking is also an important risk factor for the development of AUD, which is characterized by chronic alcohol drinking despite negative consequences and, in its more severe form, dependence on alcohol (52, 53). Thus, we further tested semaglutide in rats that were made dependent on alcohol via a well-established procedure of chronic, intermittent alcohol vapor exposure (54), and we found that semaglutide reduced dependence-induced alcohol intake, again with no sex differences. Collectively, the present findings that semaglutide suppresses different patterns of alcohol drinking (binge-like drinking in mice and rats and dependence-induced drinking in rats) provide compelling support for testing semaglutide in future clinical trials in people with AUD.

Given semaglutide’s role in reducing appetite and body weight, a critical question is whether the effects of semaglutide in reducing alcohol intake are unique to alcohol or expand to other caloric/palatable solutions. To address this question, we performed a comprehensive set of experiments in mice, using the same paradigm as alcohol (i.e., drinking-in-dark), to examine the effects of semaglutide on the consumption of non–alcohol-containing solutions that were diverse in terms of calorie content, macronutrients, and sweetness. Here, in addition to reducing alcohol binge-like drinking (with and without sweeteners), semaglutide reduced the intake of noncaloric (water and saccharin) and caloric (maltodextrin and corn oil) solutions not containing alcohol. From a mechanistic standpoint, these results suggest that semaglutide’s effects in suppressing consummatory behaviors are not specific to alcohol and might be driven by its ability to reduce appetite and thirst, such as the need for general fluid intake (55–60), palatability for sweet (taste) (61–64), and/or metabolic energy needs and calorie intake (24, 65–68). These results are not surprising, given that the role of semaglutide and other GLP-1 analogues in reducing appetite, calorie intake, and consummatory behaviors has been well documented — factors that contributed to semaglutide’s approval for the treatment of obesity (69). We believe, for at least 3 reasons, that these findings do not discount the potential for semaglutide as a pharmacotherapy for AUD. First, many medications approved, or used off-label, for the treatment of AUD also influence appetite and weight (70). For example, topiramate is known to reduce weight and is approved, combined with phentermine, for the treatment of obesity (71); although not officially approved, topiramate is recommended by the American Psychiatric Association (APA) (72) and the U.S. Department of Veterans Affairs (VA) (73) as a potential second-line treatment for AUD. Second, alcohol is often mixed with sweeteners and consumed with food; therefore, a medication like semaglutide may also help people reduce the consumption of palatable/caloric drinks and foods. Third, AUD and obesity are often comorbid with overlapping and synergistic medical consequences (e.g., liver, metabolic, and cardiovascular diseases) (74–77); therefore, semaglutide may have a dual beneficial effect by not only reducing alcohol intake but also improving other health-related outcomes.

The findings of this study also raise a long-debated question on whether the nonspecific anticonsummatory effects of semaglutide are driven by visceral malaise and/or aversion rather than attenuation of motivation to consume food or alcohol. Nausea is among the most common side effects of all GLP-1 analogues. Previous studies have shown that GLP-1R activation by exogenous GLP-1, exendin-4, or liraglutide in rodents induced conditioned taste avoidance and pica behavior that can be considered visceral malaise (78–81), though similar indicators of malaise were not observed in nonhuman primates (82, 83). Ghidewon and colleagues demonstrated that peripherally administered semaglutide both induced visceral malaise and reduced motivation for food in rats (84). Other studies suggest that the effects of GLP-1 on visceral malaise and consummatory behavior are dissociable and may be mediated by distinct populations of GLP-1Rs (26, 57, 79, 85–88). For example, exendin-4 administered into the nucleus accumbens, ventral tegmental area, and NTS reduced food and drug reward behavior (26, 86, 87, 89–97), without producing conditioned taste avoidance or pica behavior (86, 87). Furthermore, the superior effect of semaglutide on weight loss relative to other selective GLP-1 analogues cannot be attributed to greater incidence of adverse gastrointestinal events in clinical populations, and such events are often transient and associated with dose escalation (46, 98). Thus, the effects of semaglutide in the present study are likely due to a combination of malaise and reduced motivation for alcohol intake, although it is worth noting that, in patients with diabetes and/or obesity treated with semaglutide, nausea and other gastrointestinal side effects are typically transitory.

To gain a detailed understanding of the scope of semaglutide’s effects, we conducted additional experiments to examine possible interactions with alcohol pharmacokinetics, motor coordination, and locomotion. These outcomes are particularly relevant from a translational standpoint, given the increasing evidence in support of considering nonabstinence endpoints in AUD clinical trials (99, 100). While this shift has important clinical and public health implications, it also highlights the importance of ruling out drug × alcohol interactions in medication development efforts for AUD. Of most importance in this context, our experiments in mice showed no effect of semaglutide on blood alcohol levels or alcohol-induced ataxia, indicating that coadministration of semaglutide and alcohol is unlikely to cause alcohol-related pharmacokinetic or additional sedative effects. We also tested potential sedative effects of semaglutide per se (i.e., in the absence of alcohol) and found that semaglutide did not impair motor coordination in mice, yet it reduced spontaneous locomotion at the highest dose. Semaglutide did not affect spontaneous locomotion in alcohol-dependent rats. Although water intake was reduced in semaglutide-treated mice, the same effect was not observed in rats — an observation consistent with our previous preliminary work in male rats (44). Differences across species, including in drug metabolism, may explain, at least in part, the different results between mice and rats. Another possible explanation is that water was offered as the sole source of fluid for mice in a single bottle, whereas rats had water and alcohol concurrently available in a 2-lever operant condition.

In an effort to gain initial mechanistic information, we tested the effects of semaglutide on GABAergic synaptic transmission in the CeA and ILC — 2 brain areas critically involved in alcohol-related behaviors (28, 29, 101, 102). We found that semaglutide induced an increase in both CeA and ILC GABA transmission in alcohol-naive rats. These results are consistent with previous studies, conducted outside the alcohol/addiction field, showing increased GABAergic signaling in the hypothalamus (103) and hippocampus (38, 104, 105) of alcohol-naive rodents after treatment with GLP-1 or other GLP-1 analogues, which might be linked to increased intracellular cAMP levels after GLP-1R activation (39, 106, 107). However, in alcohol-dependent rats, we found mixed effects of semaglutide on GABA signaling in both CeA and ILC. Specifically, we found that semaglutide increased network-dependent GABA release in a small subset of cells, while it decreased it in the remaining cells, resulting in an average of no effect of semaglutide on GABAergic synapses in the context of alcohol dependence. Elevated GABAergic signaling in the CeA following chronic alcohol exposure is observed across multiple species (28, 33, 108, 109), and reducing the heightened GABAergic tone in the CeA is a common denominator of various drugs that suppress alcohol consumption (101, 110, 111). Based on the present electrophysiology data, we can only speculate potential mechanisms underlying the mixed effects of GLP-1R activation on CeA and ILC GABA transmission in the alcohol-dependent animals. For instance, liraglutide’s effect on GABA transmission in the hippocampus has been shown to require an intact synaptic network, as blocking the generation and propagation of action potentials abolished liraglutide-induced enhancement of GABAergic activity (104). Thus, the observed decreases of network-dependent GABA transmission with semaglutide may reflect activation of the synaptic network comprising upstream inhibitory neurons rather than a simple presynaptic effect of semaglutide on GABAergic terminals within the CeA and ILC. Alternatively, or additionally, alcohol exposure may alter intracellular mechanisms linked to GLP-1R activation resulting in opposing effects of semaglutide on distinct neuronal subpopulations that may project to different brain regions. Collectively, although our electrophysiology results do not fully explain semaglutide’s effects on alcohol intake, these data point to important neuroadaptations in the GLP-1 system and subsequent regulation of CeA and ILC GABAergic synapses in the context of alcohol dependence.

From a translational medication development standpoint, it is critical to identify potential factors that predict response to certain AUD medications (1, 112). Although the efficacy of semaglutide and other GLP-1 analogues for AUD should be demonstrated in clinical trials, it is unlikely that they will work for all people. A recent clinical trial tested the GLP-1 analogue exenatide extended-release (once weekly) in people with AUD and found that, compared with placebo, exenatide did not reduce alcohol drinking in the whole sample. However, exploratory analyses showed that exenatide significantly reduced alcohol drinking in a subgroup of patients with AUD and comorbid obesity (BMI > 30 kg/m^2^) (113). Further highlighting a potential role for GLP-1 analogues in AUD management, a recent cohort study, complemented with a self-controlled case series analysis, suggested that the use of GLP-1 analogues (grouped as a class and prescribed for their currently approved indications) might be associated with lower incidence of alcohol-related events (114).

The present set of psychopharmacological and electrophysiological data provide further support for a role of GLP-1 in alcohol drinking and other consummatory behaviors. These are translationally relevant findings and overall consistent with recent human evidence that suggest a role of the GLP-1 system in alcohol drinking and AUD, as indicated by alcohol administration studies (115, 116), postmortem brain analyses (115), and neuroimaging-genetic investigations (116, 117). Our behavioral experiments were performed in 2 species of both sexes, employed a range of alcohol-related phenotypes, and included a comprehensive set of control experiments to account for semaglutide’s potential nonspecific effects. Unlike most of the previous literature in the alcohol/addiction field, we tested a newer long-acting GLP-1 analogue, semaglutide, which has high potential for clinical translation. Our electrophysiological experiments, conducted in both alcohol-naive and -dependent rats, also provide important, yet preliminary, mechanistic information on the central effects of semaglutide and possibly other GLP-1 analogues in the context of alcohol use. An important consideration for our electrophysiology work is that future studies should expand to other brain regions and networks that are key to both alcohol consumption and GLP-1 signaling. For example, the NTS is a key region where some GLP-1 neurons show hyperexcitability after alcohol withdrawal (118). Unlike our behavioral experiments that included both sexes, the electrophysiology experiments only included males, and future work should expand to females.

In summary, this work demonstrates key biobehavioral effects of the GLP-1 analogue semaglutide in reducing alcohol drinking and modulating central GABA neurotransmission, which provide compelling support for the role of the GLP-1 system as a potential pharmacotherapeutic target for AUD.

## Methods

### Animals.

Adult, male (*n* = 40) and female (*n* = 37) C57BL/6J mice were obtained from The Jackson Laboratory and weighed between 15 and 25 g upon arrival. Adult, male (*n* = 21) and female (*n* = 21) Wistar rats were obtained from Charles River Laboratories and weighed between 180 and 360 g at the start of behavioral experiment. Adult, male (*n* = 18) Wistar rats used for electrophysiology studies were bred at The Scripps Research Institute and weighed between 380 and 700 g. Mice and rats were single and group housed, respectively, in standard cages and in separate temperature- and humidity-controlled rooms with a reverse 12-hour/12-hour light/dark cycle (22°C ± 2°C, 50%–60%, lights on at 7 p.m.). All behavioral tests were conducted during the dark cycle. Food and water were available ad libitum except during behavioral testing. Animals were habituated to the animal facilities for at least 1 week prior to starting the experiments.

### Drugs.

For behavioral testing, semaglutide (Peptide International) was prepared using 1.25% (v/v) dimethyl sulfoxide (DMSO; Thermo Fisher Scientific) and 1.25% (v/v) Tween 80 (Thermo Fisher Scientific), and diluted with 0.9% saline (Hospira). Following a within-subjects, Latin-square design, semaglutide (0.001, 0.003, 0.01, 0.03, 0.1 mg/kg in mice; 0.001, 0.01, 0.1 mg/kg in rats), and vehicle were administered s.c. The volume of injection was 10 mL/kg in mice and 1 mL/kg in rats. The alcohol solution used for systemic injections in the rotarod experiments in mice was prepared with 200-proof ethanol (Pharmco) in 0.9% saline to produce a 20% (v/v) alcohol solution. This solution was administered i.p. at a dose of 2.0 g/kg. For electrophysiology studies, stock solutions of semaglutide (BOC Sciences), CGP55845A (Tocris), 6,7-dinitroquinoxaline-2,3-dione (DNQX; Tocris), and DL-2-amino-5-phosphonovalerate (DL-AP5; Tocris) were prepared in either distilled water or DMSO, aliquoted, frozen, and added to the bath solution.

### Drinking solutions.

All drinking solutions were prepared using tap water and 190-proof ethanol (The Warner-Graham Company). Mice with access to alcohol were given either a sweet (20% v/v ethanol, 3% w/v glucose, 0.1% w/v saccharin) or unsweet (20% v/v ethanol) alcohol solution. Mice given access to drinking solutions not containing alcohol received either a sweet caloric (0.3% w/v glucose, 0.01% w/v saccharin) solution, a sweet noncaloric (0.1% w/v saccharin) solution, an unsweet carbohydrate (28% w/v maltodextrin) solution, an unsweet fat (12.5% w/v corn oil, 0.5% v/v Tween 80) emulsion, or tap water. The calorie content of the maltodextrin solution and corn oil emulsion approximates that of 20% v/v ethanol. For operant self-administration, nondependent rats were given access to a sweet alcohol solution (10% w/v ethanol, 3% w/v glucose, 0.1% w/v saccharin), and alcohol-dependent rats were given access to an unsweet alcohol solution (10% w/v ethanol).

### Drinking-in-the-dark test in mice.

A drinking-in-the-dark (DID) test was used to model binge-like drinking in mice (119, 120). Initially, a 4-day protocol was used in which mice had access to drinking solutions for 2 hours for the first 3 days, and for 4 hours on the fourth day. We adhered to this schedule for 3 weeks before switching to a modified 2-day DID procedure. Here, mice received a 2-hour session for 1 day and a 4-hour session the next day. After a day off, a second round of 2-day DID was conducted in the same week. The effects of semaglutide (2 doses per week) were evaluated during the 4-hour test sessions. Semaglutide was administered (s.c.) 30 minutes before mice were given access to the drinking solutions, 3 hours into the dark phase (119). During all DID sessions, food and water were removed from the home cages. Mice with access to only tap water during the DID session were water deprived immediately after the 2-hour DID session and were given access again during the 4-hour DID session the next day. The volume/calories consumed were calculated from weight change of drinking bottles, which were weighed at 0, 2, and/or 4 hours during a DID session.

### Food and water intake in mice.

The effects of semaglutide on chow and water intake were evaluated in mice that were previously drinking unsweet alcohol. Semaglutide or vehicle was administered 3 hours into the dark phase, and food and water were measured 24 hours after treatment.

### Motor coordination and blood alcohol levels in mice.

The effects of semaglutide on motor coordination were evaluated using an accelerating rotarod test in mice (120, 121). Mice that were previously drinking unsweet alcohol were placed on the rotarod apparatus (Rotamex-5, Columbus Instruments) and habituated for 1 minute with the rod rotating at a constant speed of 4 rpm. During training and test trials, mice were placed on the rod set at 4 rpm with a constant acceleration rate of 8 rpm/min up to a maximum of 40 rpm. The latency to fall was automatically recorded by photocell beams, with a maximum cutoff latency of 5 minutes. Immediately following habituation, mice received 5 consecutive training trials, separated by 5-minute rest intervals, and were given a minimum resting period of 24 hours prior to test trials. On testing days, mice were given 2 baseline trials separated by 5-minute rest intervals.

To test the effects of semaglutide per se on motor coordination (saline condition), mice were administered vehicle or semaglutide (0.01, 0.1 mg/kg; s.c.). Thirty minutes later, they were injected with saline (10 mL/kg; i.p.) and were tested on the rotarod 30, 60, and 90 minutes after saline injection. To test the effects of semaglutide on alcohol-induced ataxia (alcohol condition), mice were injected vehicle or semaglutide (0.01, 0.1 mg/kg; s.c.), followed by 2 g/kg alcohol 30 minutes later and were then tested on the rotarod 15, 30, 60, 90, and 120 minutes after alcohol injection. For both saline and alcohol conditions, we used a within-subjects, Latin-square design, with each testing occurring at least 24 hours apart. Blood was collected via the submandibular vein immediately after the 30- and 90-minute test trials to measure BALs, using an Analox Alcohol Analyzer (Analox Technologies North America).

### Spontaneous locomotion test in mice.

A circular corridor test (120) was used to evaluate the effects of semaglutide on spontaneous locomotion in mice that were given access to tap water during DID. The circular corridor apparatus (Thermal Gradient Ring, Ugo Basile) was at room temperature (22°C) throughout the experiment. Mice were first allowed to explore the apparatus freely for 20 minutes to habituate and were then given a 24-hour minimum rest period. On test days, mice were administered vehicle or semaglutide (0.01, 0.1 mg/kg; s.c.) in a within-subjects, Latin-square design and returned to their home-cages for 3 hours. Mice were then placed in the circular corridor for a 20-minute test session. AnyMaze Video Tracking Software (Stoelting) was used to track the total distance traveled by each mouse.

### Operant alcohol self-administration in rats.

Sweet and unsweet alcohol solutions were used for operant self-administration in rats (54, 122). To model alcohol binge-like drinking, rats were trained to self-administer a sweet alcohol (10% v/v ethanol, 3% w/v glucose, 0.1% w/v saccharin) solution and water under a free-choice, fixed ratio 1 (FR1) schedule of reinforcement in standard operant conditioning chambers (28 × 26 × 20 cm; Med Associates) (122, 123). Alcohol-dependent rats were trained similarly except that they received unsweet alcohol (10% v/v ethanol) and water (54, 120). Each operant response to the alcohol- or water-associated lever was reinforced with the delivery of 0.1 mL of fluid. Following operant responses to alcohol, a cue light located above the alcohol-associated lever was illuminated for the duration of the alcohol solution delivery (2 seconds). During this time, additional lever presses did not lead to another fluid delivery. No cue light was associated with the delivery of water. After about 16 training sessions, rats underwent 30-minute FR1 self-administration sessions to evaluate the effects of semaglutide. Semaglutide (0.001, 0.01, 0.1 mg/kg; s.c.) or vehicle was administered 3 hours prior to each self-administration session, following a within-subjects, Latin-square design.

### Alcohol vapor exposure in rats.

Rats that were trained on unsweet alcohol operant self-administration (described above) were made dependent on alcohol by chronic, intermittent alcohol vapor exposure (54, 120, 124). Briefly, rats were exposed to 14 hours of alcohol vapor per day, followed by 10 hours of room air (withdrawal). The target BAL for the rats during alcohol vapor exposure was between 150 and 250 mg/dL. Rats underwent behavioral testing during acute spontaneous withdrawal (i.e., 6–8 hours after vapor turned off). Nondependent rats were exposed to air without alcohol and were tested at the same time as the dependent rats. Semaglutide (0, 0.001, 0.01, and 0.1 mg/kg; i.p.) was administered 3 hours prior to a drinking session.

Rats used for the electrophysiology experiments were also made dependent on alcohol following an alcohol vapor protocol over 5–7 weeks (31, 32, 101). BALs were measured 1–2 times per week (average BAL = 193 ± 27 mg/dL), and air-exposed rats were used as controls (alcohol-naive).

### Spontaneous locomotion in rats.

The effects of semaglutide on spontaneous locomotion in rats were assessed using an open-field test. Alcohol-dependent rats were first habituated to the apparatus (40 × 40 cm) for 15 minutes. On testing days, rats were administered with semaglutide (0.1 mg/kg; s.c.) or vehicle, in a randomized order and, 3 hours later, were placed in the center of the open field and allowed free access for 15 minutes. The open field tests were separated by at least 3 days and conducted under red light. AnyMaze Video Tracking software (Stoelting) was used to track the total distance traveled by each rat.

### Slice preparation and electrophysiological recordings.

Preparation of brain slices and electrophysiological recordings were performed as previously described (31–33). Briefly, deeply anesthetized rats (3%–5% isoflurane anesthesia) were rapidly decapitated, and their brains were isolated in an ice-cold, oxygenated, high-sucrose cutting solution (composition in mM: 206 sucrose, 2.5 KCl, 0.5 CaCl_2_, 7 MgCl_2_, 1.2 NaH_2_PO_4_, 26 NaHCO_3_, 5 glucose, and 5 HEPES; Sigma-Aldrich). We then divided the brains with a coronal cut roughly at bregma to enable cutting acute brain slices from 2 different regions at the same time. Specifically, we cut coronal slices containing the medial subdivision of the CeA (300 μM; using a Leica VT 1000S vibratome) and coronal slices containing the ILC (300 μm; using a Leica VT1200 vibratome), which were then incubated for 30 minutes in 37°C warm, oxygenated artificial cerebrospinal fluid (aCSF) (composition in mM: 130 NaCl, 3.5 KCl, 2 CaCl_2_, 1.25 NaH_2_PO_4_, 1.5 MgSO_4_, 24 NaHCO_3_, and 10 glucose; Sigma-Aldrich), followed by another 30-minute incubation at room temperature. Dependent rats were euthanized within the last hour of their daily alcohol vapor exposure. We did not add ethanol to any of the solutions used for preparation and incubation of brain slices; thus, the slices underwent acute in vitro withdrawal, as previously shown (31, 32, 101).

Using whole-cell patch clamp, we recorded pharmacologically isolated GABA_A_ receptor–mediated sIPSCs from 33 CeA and 26 ILC neurons held at –60 mV by adding 20 μM DNQX (to block AMPA and kainate receptors), 30 μM AP-5 (to block NMDA receptors), and 1 μM CGP55845A (to block presynaptic GABA_B_ receptors) to the bath solution (31–33, 109–111). Neurons were visualized with infrared differential interference contrast optics, using either 40***×*** or 60***×*** water-immersion objectives (Olympus BX51WI, Olympus Scientific Solutions), and CCD cameras (EXi Aqua, QImaging Corporation). We did not select a specific neuronal cell type in the CeA (125), while we recorded only from pyramidal neurons in layer 5 of the ILC (capacitance > 70 pF). All recordings were performed in gap-free acquisition mode with a 20 kHz sampling rate and 10 kHz low-pass filtering, using a MultiClamp700B amplifier, Digidata 1440A, and pClamp 10 software (Molecular Devices). Patch pipettes were pulled from borosilicate glass (3–5 mΩ, King Precision Glass) and filled with a KCl-based internal solution (composition in mM: 145 KCl, 5 EGTA, 5 MgCl_2_, 10 HEPES, 2 Mg-ATP, and 0.2 Na-GTP; pH 7.2–7.4 adjusted with 1M NaOH, 295–315 mOsms; Sigma-Aldrich). We only recorded from neurons with an access resistance (Ra) < 15 MΩ, which changed less than < 20% during the recording, as monitored by frequent 10 mV pulses. Semaglutide (100 nM) (126) was applied by bath perfusion.

### Statistics.

The DID data of mice drinking sweet alcohol, unsweet alcohol, or the sweet caloric solution not containing alcohol as well as the operant self-administration data of rats (binge-like and dependence-induced drinking) were analyzed using 2-way repeated-measures ANOVA with Dose as the within-subjects factor and Sex as the between-subjects factor. Since we did not find interactions between Dose and Sex, all other behavioral data were analyzed combining males and females. Thus, the DID data of mice drinking tap water, the saccharin solution (sweet, noncaloric), the maltodextrin solution (unsweet, caloric), or the corn oil emulsion (unsweet, caloric); chow and water intake data; and spontaneous locomotion data (total distance traveled on the circular corridor and in the open field by mice and rats, respectively) were analyzed using 1-way repeated-measures ANOVA or paired 2-tailed Student’s *t* test, with Dose as the within-subjects factor. The rotarod and BAL data of mice were analyzed using 2-way repeated-measures ANOVA with Dose and Time as within-subjects factors; saline condition and alcohol condition were analyzed separately. When appropriate, Dunnett’s, Tukey’s, or Duncan’s tests were used for post hoc comparisons.

The electrophysiology data were obtained from 59 individual neurons from 18 different rats. The frequency, amplitude, rise, and decay time of sIPSCs were analyzed semiautomatically using MiniAnalysis software (Synaptosoft Inc.). Each event was visually confirmed, and sIPSCs with amplitudes < 5 pA were excluded. We combined events from 3-minute bins to obtain averaged sIPSC characteristics. To account for cell-to-cell variability, we normalized all relevant parameters (frequency, amplitude, rise, and decay time) to baseline control conditions and pooled data before group analyses. We used 1-sample *t* tests to examine changes from baseline control conditions. Unpaired Student’s *t* tests were then used to compare semaglutide effects on sIPSC characteristic between alcohol-naive and alcohol-dependent groups.

All data are represented as mean ± SEM. A *P* value less than 0.05 (2-tailed) was considered significant. Prism 8 (GraphPad Prism) and Statistica 13 (TIBCO Software) were used for the analyses.

### Study approval.

All procedures were performed according to the *Guide for the Care and Use of Laboratory Animals* (National Academies Press, 2011) and were approved by the IACUC of the NIDA IRP or The Scripps Research Institute.

### Data availability.

The data are available from the corresponding authors upon reasonable request.

## Author contributions

VC, MF, SK, MR, LFV, and LL conceptualized the study and designed the experiments. VC, SK, CLP, SKE, RV, and RCNM conducted the experiments. VC, MF, SK, CLP, RV, RCNM, and LFV analyzed the data. VC, MF, SK, CLP, MR, LFV, and LL wrote the original draft. RCNM and GFK provided critical feedback. MF, GFK, MR, LFV, and LL provided funding. All authors read and approved the final manuscript. The 3 co–first authors (VC, MF, and SK) were listed in alphabetical order.

## Supplementary Material

Supplemental data

## Version 1 05/16/2023

In-Press Preview

## Version 2 06/22/2023

Electronic publication
