# Supplementary material for: The glucagon-like peptide-1 (GLP-1) analogue semaglutide reduces alcohol drinking and modulates central GABA neurotransmission
Source: JCI Insight. 2023 Jun 22;8(12):e170671. doi: 10.1172/jci.insight.170671 (PMC10371247; doi:10.1172/jci.insight.170671)
Supplement: Supplemental data [file jciinsight-8-170671-s142.pdf]

Supplementary Information

Chuong V^, Farokhnia M^, Khom S^, Pince CL, Elvig SK, Vlkolinsky R, Marchette RCN, Koob GF, Roberto M*, Vendruscolo LF*, Leggio L*. The glucagon-like peptide-1 (GLP-1) analogue semaglutide reduces alcohol drinking and modulates central GABA neurotransmission.
^co-first author, *co-senior author

Table of Contents:

- **Table S1.** Semaglutide reduced chow and water intake in mice.

- **Figure S1.** Semaglutide reduced spontaneous locomotion but had no effect on motor coordination, alcohol-induced ataxia, or blood alcohol levels in mice.

- **Figure S2.** Chronic intermittent ethanol vapor exposure enhanced GABA neurotransmission in central nucleus of the amygdala (CeA) and infralimbic cortex (ILC) neurons from male rats.

- **Table S2.** Comparison of baseline spontaneous inhibitory postsynaptic currents (sIPSC) characteristics in (A) central nucleus of the amygdala (CeA) and (B) infralimbic cortex neurons (ILC) neurons between alcohol-naïve and alcohol-dependent male rats.

Table S1. Semaglutide reduced chow and water intake in mice.

Treatment	Chow (g/kg)	Water (mL/kg)
**Vehicle**	139.9 ± 7.4	146.6 ± 9.5
**Semaglutide, 0.001 mg/kg**	122.5 ± 8.2	97.4 ± 9.5 (***)
**Semaglutide, 0.003 mg/kg**	93.1 ± 7.2 (****)	98.6 ± 6.3 (***)
**Semaglutide, 0.01 mg/kg**	66.2 ± 7.4 (****)	73.1 ± 6.1 (****)
**Semaglutide, 0.03 mg/kg**	39.6 ± 5.6 (****)	48.1 ± 6.7 (****)
**Semaglutide, 0.1 mg/kg**	38.2 ± 7.9 (****)	45.8 ± 6.7 (****)

Chow and water intake were examined in mice that were previously drinking unsweet alcohol (n = 7 males and 8 females). All data are expressed as mean ± SEM and were analyzed using one-way repeated-measures ANOVAs. ***$p < 0.001$, ****$p < 0.0001$, *vs.* Vehicle.

Figure S1. Semaglutide reduced spontaneous locomotion but had no effect on motor coordination, alcohol-induced ataxia, or blood alcohol levels in mice.

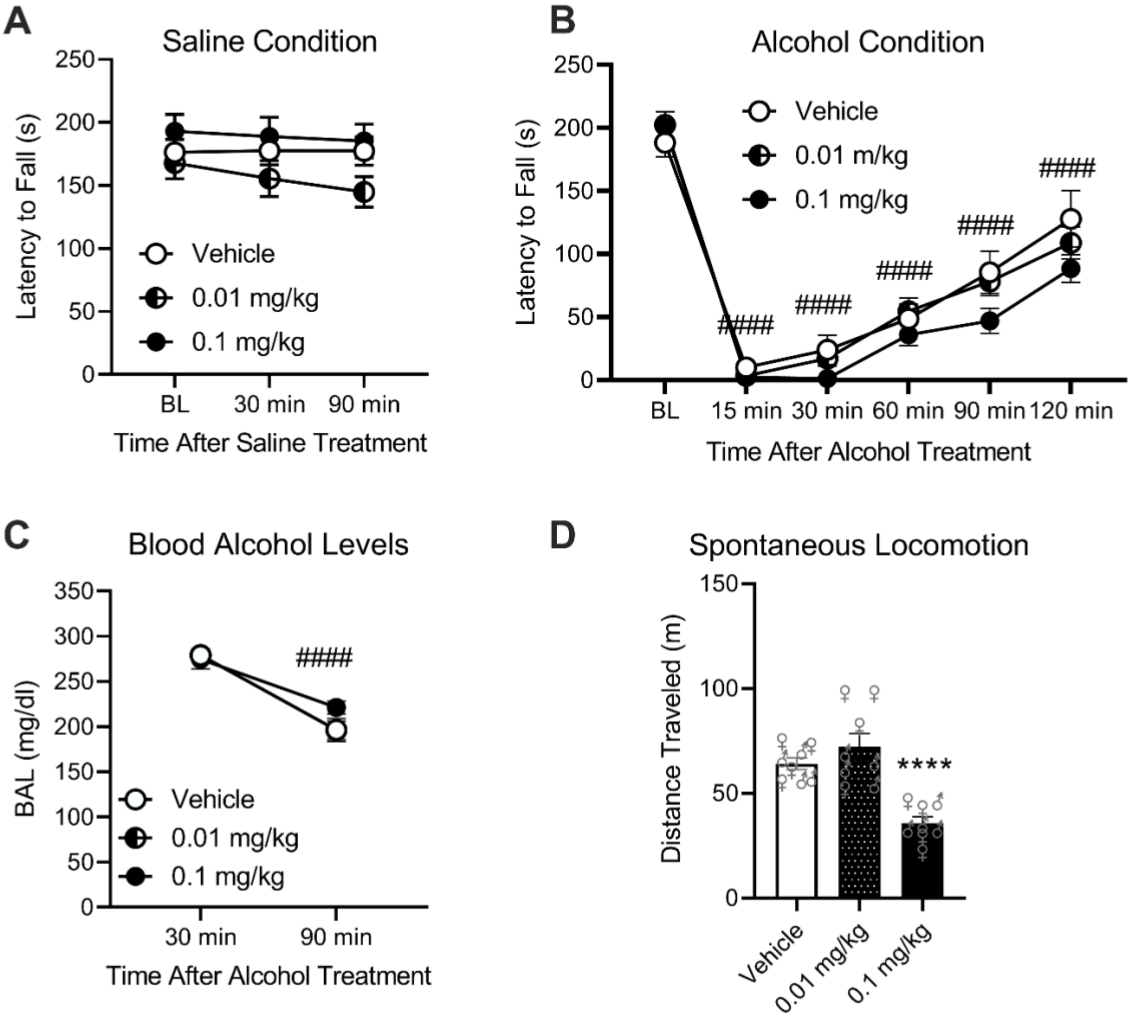

(**A**) Semaglutide did not disrupt motor coordination in mice. (**B**) Intraperitoneal alcohol administration resulted in significant motor impairment (i.e., alcohol-induced ataxia) in mice. Semaglutide had no effect on alcohol-induced ataxia on the rotarod. (**C**) Semaglutide had no effect on blood alcohol levels (BALs) 30 min and 90 min after intraperitoneal alcohol administration. Males ($n = 8$); females ($n = 7$). (**D**) Semaglutide at 0.1 mg/kg decreased spontaneous locomotion in the circular corridor as measured by the total distance traveled. Males ($n = 4$); females ($n = 7$). ****$p < 0.0001$, *vs.* Vehicle. #### $p < 0.0001$, different from baseline (BL; A and B) and 30 min (C). Data are expressed as mean ± SEM and were analyzed using one- or two-way repeated-measures ANOVAs.

Figure S2. Chronic intermittent ethanol vapor exposure enhanced GABA neurotransmission in central nucleus of the amygdala (CeA) and infralimbic cortex (ILC) neurons from male rats.

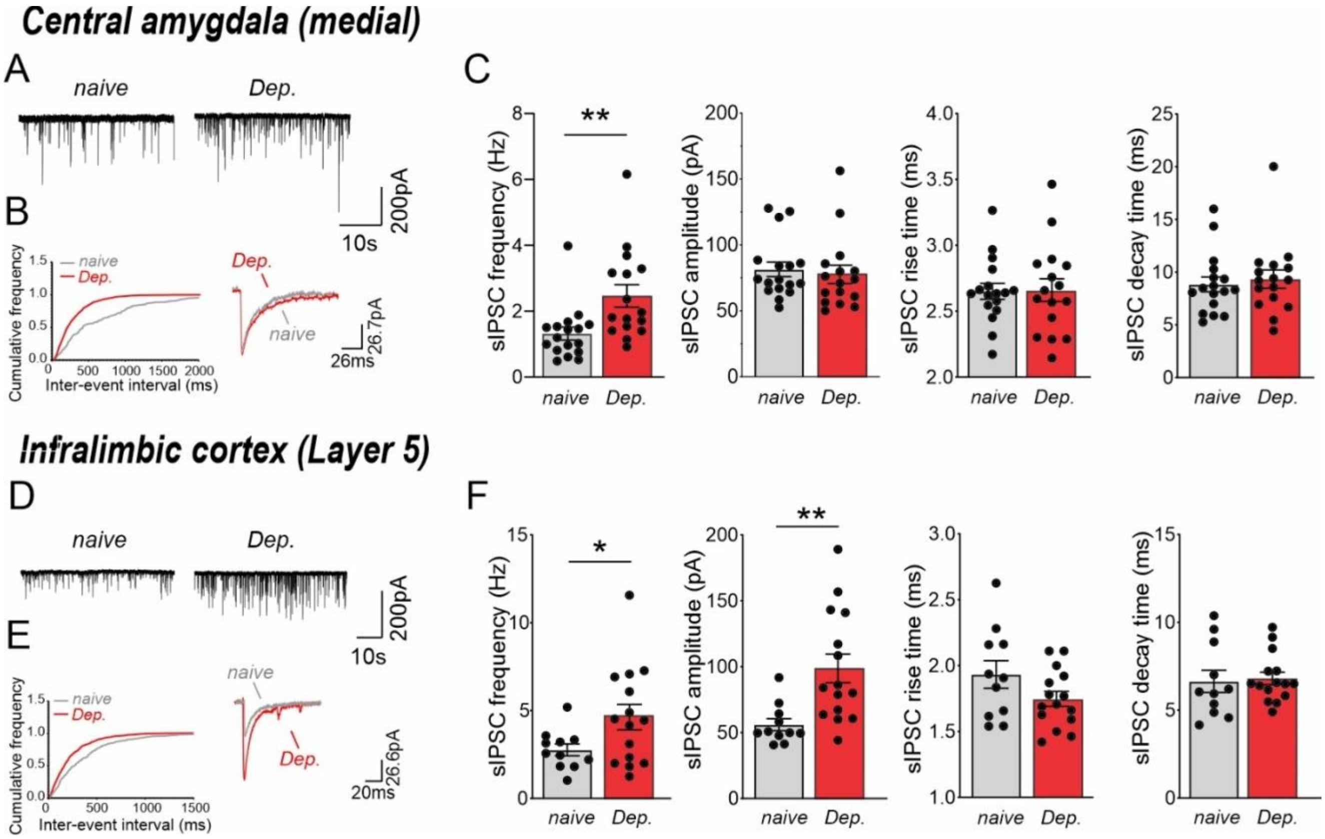

(**A**) Representative spontaneous inhibitory postsynaptic currents (sIPSC) traces of central nucleus of the amygdala (CeA) neurons. (**B**) cumulative frequency distributions and scaled sIPSC averages from the traces depicted in (**A**) for CeA neurons of alcohol-naïve or alcohol-dependent rats. (**C**) Mean ± SEM of sIPSC frequencies, amplitudes, rise, and decay times in CeA neurons of alcohol-naïve or alcohol-dependent rats. (**D**) Representative sIPSC traces of infralimbic cortex (ILC) neurons. (**E**) cumulative frequency distributions and scaled sIPSC averages from the traces depicted in (**D**) for ILC neurons of alcohol-naïve or alcohol-dependent rats. (**F**) Mean ± SEM of sIPSC frequencies, amplitudes, rise, and decay times in ILC neurons of alcohol-naïve or alcohol-dependent rats. Data were generated from 16-17 CeA and 11-15 ILC neurons. *$p < 0.05$, **$p < 0.01$, alcohol-naïve *vs.* alcohol-dependent; unpaired Student's *t*-tests (two-tailed). Alcohol-naïve rats ($n = 8$), alcohol-dependent rats ($n = 10$).

Table S2. Comparison of baseline spontaneous inhibitory postsynaptic currents (sIPSC) characteristics in (**A**) central nucleus of the amygdala (CeA) and **(B)** infralimbic cortex neurons (ILC) neurons between alcohol-naïve and alcohol-dependent male rats.

**(A) CeA**	**Alcohol-naïve**	**Alcohol-dependent**
**Number of cells (n)**	17	16
**Frequency (Hz)**	1.32 ± 0.20	2.46 ± 0.34 (**)
**Amplitude (pA)**	81.44 ± 5.53	77.67 ± 6.96
**Rise time (ms)**	2.65 ± 0.06	2.66 ± 0.09
**Decay time (ms)**	8.84 ± 0.70	9.34 ± 0.87
**(B) ILC**	**Alcohol-naïve**	**Alcohol-dependent**
**Number of cells (n)**	11	15
**Frequency (Hz)**	2.76 ± 0.34	4.63 ± 0.72 (*)
**Amplitude (pA)**	56.03 ± 4.55	98.71 ± 10.91 (**)
**Rise time (ms)**	1.93 ± 0.10	1.75 ± 0.06
**Decay time (ms)**	6.64 ± 0.63	6.80 ± 0.35

*$p < 0.05$, **$p < 0.01$, alcohol-naïve *vs.* alcohol-dependent; unpaired Student's *t*-tests (two-tailed). Alcohol-naïve rats ($n = 8$), alcohol-dependent rats ($n = 10$).
